# The complicated history of the intricate relationship of posttraumatic fissures, fractures, and intracranial hematomas in neurotraumatology

**DOI:** 10.1007/s00701-023-05660-0

**Published:** 2023-06-30

**Authors:** R. Firsching

**Affiliations:** Universitätsklinik Für Neurochirurgie, Leipzigerstr. 44, 39120 Magdeburg, Germany

Trepanation is the earliest surgical activity of man we have proof of. According to the findings in the necropolis of Taforalt [1], trepanation was performed at least 10,000 years ago. The information given on the indication for trepanation for posttraumatic fissures by prominent pioneers dates to Hippocrates. The uncertainty as to the handling of “fissures,” or depressed skull fractures, after head injury has been presented with its opposing indication, technique, and urgency of trepanation for over 2000 years. We must be grateful for this careful and detailed account of our history.

Some of the opinions and conclusions expressed in this article are debatable. It is claimed that “in the time before computerized tomography, fissures were of interest simply as an indicator of the severity of the cranial trauma which produced them.” This claim is not quite precise, as the identification of a fissure has a practical effect recognized today: When x-ray confirms a fissure, for example, in the absence of available computerized tomography, a close clinical monitoring is mandatory according to several guidelines. Prior to the era of computerized tomography, the extradural hematoma could then be identified or ruled out with ultrasound or angiography. Discharging a patient with an unrecognized acute fracture after head injury remains a regrettable cause of fatal outcomes.

Petit (1674–1750) published on this topic, and there is evidence that sizable volumes were published, as German translations between 1725 and 1743 [2]. These are still on sale on the public book market. After his death, his lectures on injuries were published between 1774 and 1790. He was the first ever to teach on the phenomenon of posttraumatic epidural hematoma underlying the fissure, the space of the “detached dura,” which had been previously described by Galen and gave a detailed description of its cause, the typical lucid interval initially following the injury, and the subsequent loss of consciousness and outlining the proper surgical treatment [3].

The historical account mentions “material extruded between the skull and the dura” as a historical indication for trepanation. Obviously, in the absence of imaging, the confirmation of the diagnosis of “detached dura” and of this “material” itself required trepanation. Thus, trepanation was always necessary, whenever this “material” was suspected. From today’s point of view, the only “material” between skull bone and dura in the acute phase after a head injury could have been blood. The ancient pioneers Galen, Paul of Egina, Borgononi, and Berengario, as each reported on the “detached dura,” had in fact exposed an epidural hematoma. So, we can assume that epidural hematomas were removed in ancient times. At that time the indication to remove an epidural hematoma was to prevent infection. This indication has become obsolete, but to relieve mass effect from the brain is now undoubtedly mandatory. So, it may be that the ancient pioneers performed the lifesaving decompression upon a misled intent, keeping in mind that the decision to perform surgery at all in ancient times was associated with high-risk infection, death, and disability.

The views communicated by Pott are rated to have been the death knell for the practice of prophylactic trepanation per se. Pott promoted the arguments that it is a lesion of the brain and not a fissure that causes symptoms like … vomiting, loss of sense, speech … and secondly that epidural blood was not the origin of the formation of pus. Both arguments were not new as we can conclude from the information given by earlier pioneers in this paper. Prior to Pott, Petit had claimed that disorder of sense, speech, and motion was not caused by a lesion of the bone but by a lesion of the brain. Lanfranc, followed by Mondeville, antedated Pott’s views by four centuries as he “rejected trepanation because he contended pus did not form following fissures.” The advances in the practice of post-traumatic surgery for fissures had not been promoted much by Pott, reiterating views from earlier pioneers. It was Petit who recommended timely removal of the hematoma under the fissure, while the fissure itself did not need much attention. With the teachings of Petit, we came to know that targeted trepanation in the area of the fissure or fracture would allow for the timely removal of an extradural hematoma under the fissure or fracture, which could be lifesaving. This was the Copernican turnaround of neurosurgical management of head injuries. Krönlein studied the preferred location of the epidural hematomas and recommended a systematic scheme of prophylactic burr holes with the greatest likelihood of exposing the hematoma under the bone [4]. This had been adopted as a popular type of prophylactic trepanation in the first half of the twentieth century and may still be relevant when proper imaging is not available.

It is quite true that progress had been stalled and even resisted within the medical science. In terms of personal persecution suffered and, patients’ lives saved Semmelweis deserves to be mentioned. The 85-year-old Nobel laureate of physics, Max Planck, saw this aspect not only restricted to medicine. He commented “I had thought a new scientific finding based on convincing evidence would gradually be understood and accepted by the scientific community. I had to reach the age of 80 years to realize this never happens. A new scientific truth will not prevail by convincing the opponents to a point they accept it, but rather by waiting until these opponents get extinct and the new generation will get used to the truth” [6].

While we criticize colleagues for obstructing progress for 2000 years, we should adequately consider the difficulties of perceiving the relative significance of post-traumatic fissures in the absence of imaging. Not before the 1980s did we understand the intricate relationship of post-traumatic fissures and fractures with intracranial hematomas. Extensive studies with computerized tomography after head injury revealed that three out of four patients with fissures recovered well without treatment. One out of four patients with a fissure or fracture will develop a surgically relevant intracranial hematoma. On the other hand, only one out of 6000 patients will develop a hematoma in the absence of a fracture [5]. Patients with severe sequelae or fatal outcomes after head injury in historical times were almost invariably found to have a fissure or fracture of the skull; so, our early pioneers were led to believe that fissures and fractures themselves were the cause of unfavorable outcomes. In the absence of modern imaging and knowledge of the pathophysiology of compression of the brain, nobody could have guessed that the frequency of fissures is three times as high in patients with favorable outcomes as in severe outcomes. Today, posttraumatic fractures still require trepanation for an underlying hematoma in about 25% of cases [5].
